# CircSI-SSL: circRNA-binding site identification based on self-supervised learning

**DOI:** 10.1093/bioinformatics/btae004

**Published:** 2024-01-05

**Authors:** Chao Cao, Chunyu Wang, Shuhong Yang, Quan Zou

**Affiliations:** Yangtze Delta Region Institute (Quzhou), University of Electronic Science and Technology of China, Quzhou, Zhejiang 324003, China; Institute of Fundamental and Frontier Sciences, University of Electronic Science and Technology of China, Chengdu, Sichuan 611731, China; Faculty of Computing, Harbin Institute of Technology, Harbin, Heilongjiang 150001, China; Faculty of Mathematics and Computer Science, Guangdong Ocean University, Zhanjiang, Guangdong 524088, China; Yangtze Delta Region Institute (Quzhou), University of Electronic Science and Technology of China, Quzhou, Zhejiang 324003, China; Institute of Fundamental and Frontier Sciences, University of Electronic Science and Technology of China, Chengdu, Sichuan 611731, China

## Abstract

**Motivation:**

In recent years, circular RNAs (circRNAs), the particular form of RNA with a closed-loop structure, have attracted widespread attention due to their physiological significance (they can directly bind proteins), leading to the development of numerous protein site identification algorithms. Unfortunately, these studies are supervised and require the vast majority of labeled samples in training to produce superior performance. But the acquisition of sample labels requires a large number of biological experiments and is difficult to obtain.

**Results:**

To resolve this matter that a great deal of tags need to be trained in the circRNA-binding site prediction task, a self-supervised learning binding site identification algorithm named CircSI-SSL is proposed in this article. According to the survey, this is unprecedented in the research field. Specifically, CircSI-SSL initially combines multiple feature coding schemes and employs RNA_Transformer for cross-view sequence prediction (self-supervised task) to learn mutual information from the multi-view data, and then fine-tuning with only a few sample labels. Comprehensive experiments on six widely used circRNA datasets indicate that our CircSI-SSL algorithm achieves excellent performance in comparison to previous algorithms, even in the extreme case where the ratio of training data to test data is 1:9. In addition, the transplantation experiment of six linRNA datasets without network modification and hyperparameter adjustment shows that CircSI-SSL has good scalability. In summary, the prediction algorithm based on self-supervised learning proposed in this article is expected to replace previous supervised algorithms and has more extensive application value.

**Availability and implementation:**

The source code and data are available at https://github.com/cc646201081/CircSI-SSL.

## 1 Introduction

Circular RNA (circRNA) is the peculiar class of RNAs produced by pre-mRNA. Unlike common RNAs with the two ends of 5′ and 3′, circRNA has a unique ring structure formed by the reverse splicing mechanism (Bogard *et al.* 2018, Hao *et al.* 2019), widely present in human, hippocampus, mouse, and other cells and tissues (Dori *et al.* 2019, Li and Han 2019). This special structure can enhance the stability of circRNA and usually has a stage-specific expression pattern (Rybak-Wolf *et al.* 2015). More and more evidence has proved that circRNA can participate in the processes of gene expression regulation through combining the corresponding RNA-binding protein (RBP) (Chen 2016, Zang *et al.* 2020). Like other non-coding RNAs (Huang *et al.* 2022a,b), It can also play a crucial part in the screening and therapy of many diseases (Jiao *et al.* 2021, Wang *et al.* 2021), especially cancer (Zhang *et al.* 2018, Su *et al.* 2022). Therefore, the understanding of the action mechanism between circRNA and RBP is crucial to reveal the circRNA formation and its biological function (Chen *et al.* 2022, Niu *et al.* 2022a,b,c).

With the emergence of some biological technologies about sequencing, such as high-throughput sequencing with crosslinking immunoprecipitation (HITS-CLIP), many RBP targets in mature circRNAs have been found in eukaryotes (Dudekula *et al.* 2016, Ruan *et al.* 2019). However, due to the high cost of detecting each pair of interaction sites, many computational methods for identifying circRNA–RBP sites have been developed. Thanks to the advancements in deep learning, the identification performance of RBP-binding sites has been continuously improved. For example, CSCRSites (Wang *et al.* 2019a,b) is a deep learning algorithm that identifies RBP-binding sites about cancer-specific only using nucleotide sequences information. CircSLNN (Ju *et al.* 2019) is a novel approach that transforms the RNA-binding site prediction problem into a sequence labeling problem, which adopts a word-embedded based coding scheme to capture the context and semantic information of sequences. CRIP (Zhang *et al.* 2019) proposes a stacked codon encoding deep learning algorithm based on convolutional neural networks and recurrent neural networks, which respectively learn abstract features and sequence dependences to complete the RBP-binding site recognition task. However, these methods are single-view algorithms, and the useful features obtained from the sequence are quite limited, and often constrained by the size of the data, and cannot achieve good performance. Subsequently, researchers have introduced some multi-view algorithms. PASSION (Jia *et al.* 2020) is a multi-view integrated neural network algorithm, and the optimal feature subset is selected and input into the network through incremental features selection and XGBoost algorithm. iCircRBP-DHN (Yang *et al.* 2021) proposes to use two new encoding schemes: K-tuple nucleotide frequency patterns and CircRNA2Vec word embedding encoding as inputs. Deep multi-scale residual network, bidirectional gate recurrent unit (BiGRU), and self-attention mechanism are used as algorithms for deep network architecture. CRBPDL (Niu *et al.* 2022a,b,c) proposes an Adaboost integrated deep network architecture, which includes deep multi-scale residual networks and BiGRU. The performance of the algorithm is further improved. HCRNET (Yang *et al.* 2022) incorporates a fine-tuned DNABERT model and a deep temporal convolutional network to capture global context-dependent semantic and syntactic information for circRNA sequences.

As for the networks based on CNN, RNN or their deformation used in the above research as deep feature extraction networks, there are problems, such as poor network parallel capability, difficulty to capture features long-time series dependence, and insufficient algorithm stability. CircSSNN (Cao *et al.* 2023) proposes an algorithm that fully uses the self-attention mechanism to extract deep features and achieves better performance. Although these algorithms are constantly updating the performance of the recognition task, they are based on supervised learning, in other words, the algorithm requires a great number of sample labels in network training. Usually, the ratio of training samples to test samples is as high as 80%:20%. Although the algorithm achieves good performance, it greatly limits the exploration of the unknown circRNA–RBP interaction mechanism. As a consequence, it has immense practical significance to develop an algorithm based on supervised weakly, self-supervised, and even unsupervised in this task.

Self-supervised learning (SSL) (Liu *et al.* 2021) is a special kind of unsupervised learning. It learns required features without the need for real labels through pre-designed agent tasks, and subsequent tasks often require only a few labels (or even none) to significantly enhance performance according to specific tasks. Contrast learning performs particularly well in computer vision because it can learn invariant representations from enhanced data without label information (Hjelm *et al.* 2019, Chen *et al.* 2020, He *et al.* 2020), demonstrating significant self-supervised capabilities. The specific operation process is as follows: first, data augmentation is used to get a number of different perspectives (usually two) from the original image that are slightly different. Then, different views of the same sample are taken as positive sample pairs and the others as negative samples. By maximizing the similarity between positive sample pairs and minimizing the similarity between positive sample and negative sample, a “label” is artificially constructed to guide the learning of network features. However, while contrast learning can be useful in the field of images, it is difficult to apply to time-series data for several reasons: above all, there exists a challenge of capturing temporal dependencies in the data, which is very critical. Secondly, image-based augmentation techniques, such as random cropping, do not work with time-series datasets. Thus far, there have been few studies on contrast learning for time-series data, and it has not been applied to the prediction of circRNA-binding sites.

For the sake of reducing the dependence of the algorithm on the sample label as much as possible, thereby enhancing its applicability across a wider range of scenarios. This article carried out in-depth research and innovatively proposed an algorithm named circRNA-binding site identification based on self-supervised learning (CircSI-SSL). The algorithm uses only KNFP, CircRNA2Vec, and electron–ion interaction pseudo-potential (EIIP) shallow statistical feature descriptors, which reduces the computational resource requirements. After encoding, our Transformer model RNA_Transformer, which is improved for CircRNA recognition task, is used to: (i) perform cross-view sequence prediction tasks to train the network, capture temporal dependencies in sequence multi-view data, and learn the overall representation of the sequence; (ii) apply a very small number of sample tags (10%) to fine-tune network parameters for a specific task, thereby completing the RBP-binding site prediction task. Through a comprehensive experiment conducted on 12 widely used datasets, it is shown that the algorithm obtains a significant improvement over the supervised learning algorithms. In summary, the primary contributions of this article can be outlined as follows.

A novelSSL method is applied in the domain of circRNA-binding protein recognition, which changes the situation that most of the label information is needed to obtain good performance. Using only a small amount of supervised information can lead to a substantial enhancement in the algorithm’s performance, which has a wide range of application value.

We propose a novel proxy task that captures sequence temporal dependencies using an improved RNA_Transformer as a benchmark model and completes cross-view sequence prediction based on multiple feature descriptors instead of using sequence augmentation techniques.

Comprehensive experiments conducted on six widely used circRNA datasets and six linRNA datasets demonstrate that the proposed algorithm exhibits comprehensive advantages over previous supervised learning approaches. Even when utilizing only 10% of the labeled data for training, the proposed algorithm demonstrates stable and outstanding performance, along with robust scalability.

## 2 Materials and methods

### 2.1 Datasets

In order to assess the validity of our approach, we selected six widely used circRNA datasets, WTAP, FXR1, C17ORF85, QKI, TAF15, and AUF1. These circRNA sequences derive from circRNA interaction set of database (https://circinteractome.nia.nih.gov/), which extracted data includes circRNA–RBP interaction information, also includes RBPs that bind to mature circRNA upstream and downstream flanker sequences (Yee *et al.* 2019). We then use the identical data processing steps as previous research (Zhang *et al.* 2019). Resulting 101 nucleotides sequence fragments in length are obtained as positive samples, and randomly selecting other sequences to acquire negative samples with same numbers. These similar sequences are removed using CD-HIT technique, with the threshold of 0.8 (Li and Godzik 2006). After the removal of sequence redundancy, a total of 15 570 samples were obtained, and all samples used in the experiments were randomly shuffled.

In addition, we transplant the circSI-SSL algorithm to linear RNA datasets and compare the performance of several existing supervised algorithms in identifying RBP interactions. The same linear RNA datasets are downloaded from iDeepS (Pan *et al.* 2018) and DeepBind (Alipanahi *et al.* 2015), including six datasets after HITS-CLIP processing: hnRNPC-2, U2AF65, hnRNPC-1, QKI, ELVAL1-2, and Y2AF65.

### 2.2 Feature muti-descriptors

To enrich the originally single sequence, we employ three quantitative feature methods to extract the preliminary statistical features of the sequence: (i) KNFP, which is used to capture local semantic features at disparate positions. (ii) CircRNA2Vec, which is employed to capture remote dependencies. (iii) The EIIP, which is utilized to characterize the free electron energy on the circRNA sequence.

#### 2.2.1 KNFP

In this section, we introduce KNFP schema in detail. Different from the traditional One-hot representation (Zhang *et al.* 2019), KNFP schema can extract various short-range sequence-dependent information (Orenstein *et al.* 2016) and local semantic features, which greatly makes up for the deficiency of One-hot information and retains the original sequence schema.

Taking a specific circRNA sequence of length L as an example, KNFP slidingly selects *k* consecutive nucleotides on the circRNA sequence, and counts the frequencies of the corresponding combinations in the form of *k* tuples (different combinations of *k* nucleotides), as the final encoding. In detail, for a *k*-tuple, which has $4^{K}$ different combinations, the frequency p of the corresponding *K*-tuple pattern is statistically calculated according to the specific circRNA sequence.

(1) $$p=\left[p_{1},p_{2},p_{3},p_{4},\ldots ,p_{4^{K}}\right].$$

Here, $p_{i}\;$represents the frequency of the i-th *k*-tuple pattern. Upon processing a single circRNA sequence, the resulting feature dimension becomes $\left(L-k+1,\;4^{K}\right)$. We concatenate the encoded features obtained by *k* = 1, 2, and 3, respectively, and complete them with 0 at the end.

#### 2.2.2 CircRNA2Vec

CircRNA2Vect (Yang *et al.* 2021) is a feature descriptor that employs the Doc2Vec algorithm to learn global contextual features of circRNA. Doc2Vec (Le and Mikolov 2014) is an extension of Word2Vec, capable of learning fixed-length feature representations from variable-length texts. Unlike Word2Vec, Doc2Vec introduces an additional paragraph vector d at the input layer, which captures the contextual information of paragraphs. This enables the linkage of word vectors with paragraph vectors, addressing the limitation of Word2Vec that focuses solely on training word vectors while overlooking the grasp of paragraph-level context.

We collect as many circRNA splicing sequences as possible from circBase (Glažar *et al.* 2014) to serve as the corpus. Utilizing a sliding window of size 10, extract subsequences from each circRNA sequence, resulting in multiple sequences. This allows the algorithm to capture semantic information within these subsequences for modeling purposes. Given a text sequence of length T, where the word at time step *t* is denoted as $w_{t}$. For context window size k, the likelihood function of the model is the probability of generating a specific word $w_{t}$, which express as term $p(w_{t}|w_{t-k},\ldots ,w_{t+k},\;d)$. The goal of the model is to maximize the average logarithmic probability as follow:

(2) $$\frac{1}{T}\sum_{t=k}^{T-k}\;\text{log}\;p\left(w_{t}|w_{t-k},\ldots ,w_{t+k},d\right).$$

#### 2.2.3 EIIP

EIIP, as introduced by Nair and Sreenadhan (2006), is a novel feature encoding scheme that describes the energy of delocalized electrons in amino acids and nucleotides present in circRNA sequences. Four binary indicator sequences are used to encode the sequence. It has been widely utilized in the Resonance Recognition Model. The EIIP values for the nucleotide “G,” “C,” “T,” and “A” are “0.0806,” “0.1340,” “0.1335,” and “0.1260,” respectively. To enrich the feature representation, we incorporate a PSTNPss encoding scheme. It is position-specific feature encoding based on single-strand of DNA. See He *et al.* (2018) for more details.

### 2.3 CircSI-SSL algorithm architecture

**Figure 1.**
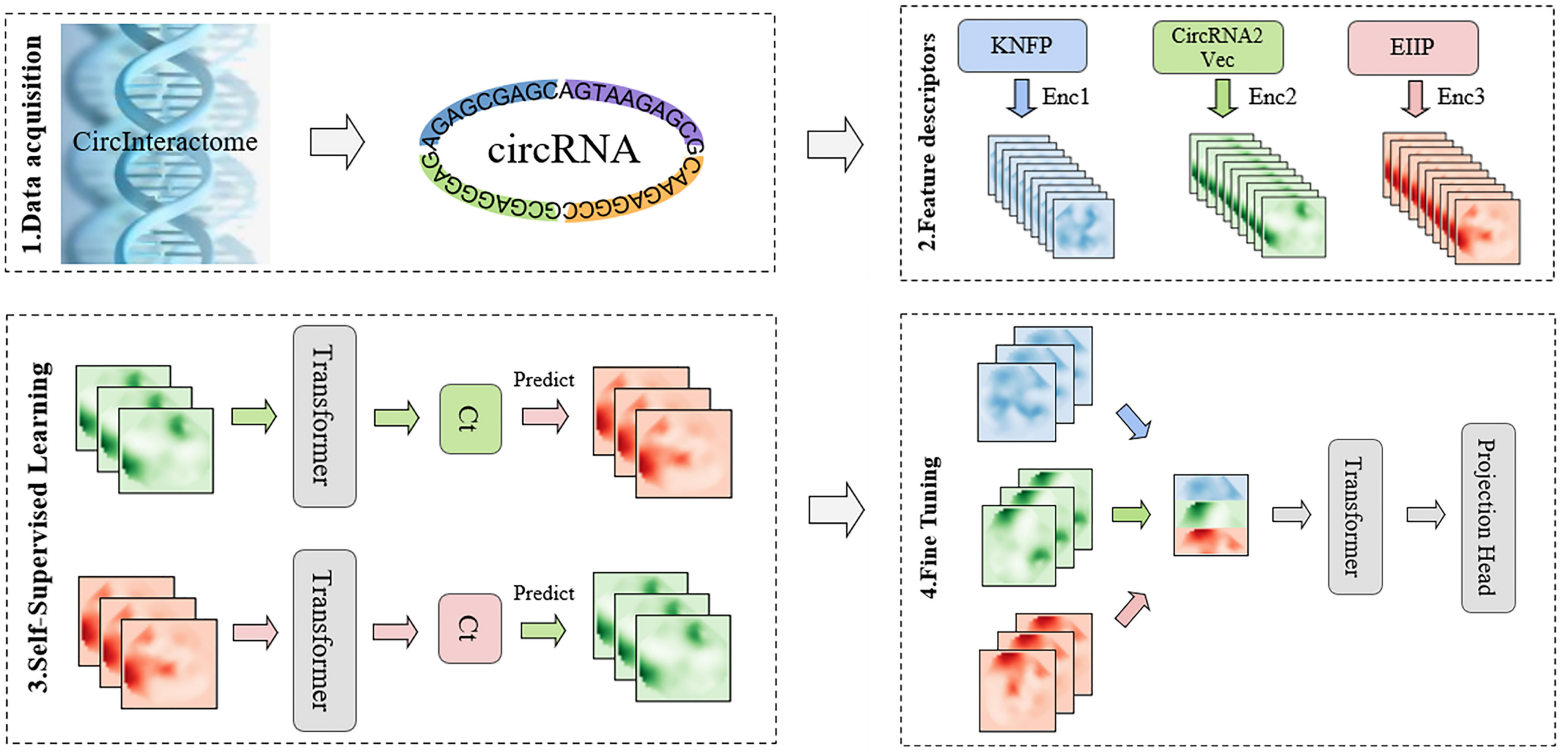
CircSI-SSL framework.

In this section, we introduce the CircSI-SSL self-supervised algorithm framework for learning high-quality representations of sequences, using only a small number of samples to fine-tune the CircSI-SSL algorithm for specific tasks to achieve excellent results. The overall framework is shown in Fig. 1. For a more intuitive understanding, we provide the pseudo-code as follow. The model consists of two components: cross-view prediction and fine-tuning. (i) Multiple feature encoders are employed to encode initial features obtained from various descriptors extracted from the raw sequence data. A cross-view sequence prediction is conducted using RNA_Transformer. (ii) The trained encoded features are then fused, followed by employing RNA_Transformer to extract structured features from the fused multi-view features based on a small number of labels, facilitating the classification task.

Algorithm 1 CircSI-SSL

**Input:** CircRNA sequence x, label y, Maximum iterations MaxIter

**Output:** Neural network parameters W, Prediction label y'

1.  for iter in range(MaxIter): # Self-supervised stage

2.  enc1 = KNFP(x)

3.  enc2 = CircRNA2Vec(x)

4.  enc3 = EIIP(x)

5.  c1 = RNA_Transformer(enc1) # Context c1 of enc1 is extracted by RNA_Transformer

6.  c2 = RNA_Transformer(enc2)

7.  c3 = RNA_Transformer(enc3)

8.  Calculate cross-view contrast loss according to Formula 6 and 7

9.  Update W according to Adam optimizer

10.  for iter in range(MaxIter): # Fine-tuning stage

11.  enc = concatenate([enc1, enc2, enc3]) # Obtaining the enc1, enc2, enc3 follows the same steps as above

12.  c = RNA_Transformer(enc)

13.  y’ = softmax(c)

14.  Calculate cross-entropy loss of y and y' according to Formula 9

15.  Fine-tune W according to Adam optimizer

#### 2.3.1 Cross-view prediction

The initial research on SSL started with the application of agent tasks on image datasets, which aims to learn high-quality representations. For example, some previous work predicts image rotation (Gidaris *et al.* 2018), images colorization (Zhang *et al.* 2016), and puzzle solving (Noroozi and Favaro 2016). By using image augmentation technology to construct positive and negative samples, the application range of contrast learning is broadened: case discrimination fields, such as SimCLR (Chen *et al.* 2020) and MoCo (He *et al.* 2020); time-series analysis, such as CPC (Oord *et al.* 2018) and TS-TC (Eldele *et al.* 2021). Unfortunately, these algorithms’ performance depends heavily on the augmentation techniques used, especially for time-series data, and it is difficult to find a set of effective and widely used augmentation techniques for operations, such as random cropping and image graying. This greatly restricts the application of contrast learning to time-series data. Building upon this, this article studies a new contrast task, which extracts features from multiple real views for mutual prediction without the help of augmentation techniques.

**Figure 2.**
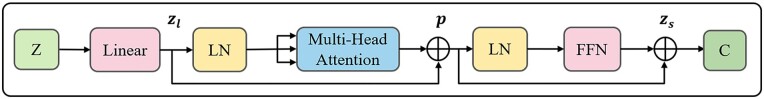
RNA_Transformer structure.

We take the improved Transformer (Vaswani *et al.* 2017) and TS-TC (Eldele *et al.* 2021) as feature extraction networks, as revealed in Fig. 2. It principal consists of Multi-head Attention and Feed Forward Neural Network (FFN), Layer Normalization (LN) blocks. The FFN block consists of a fully connected layer, a non-linear ReLU function and dropout. The model uses a pre-norm residual connection (Wang *et al.* 2019a,b) and LN prior to passing through a multi-head self-attention network, resulting in more stable gradients:

(3) $${\mathrm{LN}}_{\gamma ,\beta }\left(z\right)=\frac{z-\mu }{\sqrt{\sigma^{2}+\epsilon }}⊙\gamma +\beta ,$$

(4) $$\text{MHA}(K,V,Q)=\mathrm{softmax}\left(\frac{QK^{T}}{\sqrt{d}}\right)V.$$

Where μ and σ are the mean and variance of z, respectively, γ and β represent the parameter vectors of scaling and translation, respectively. Query information, key information, and value information related to a specific task are represented as q, k, and v, respectively. The number of operation heads is represented as h, and the aggregated q, k, and v after multiple heads are denoted as Q, K, and V, respectively. Here, d signifies the dimensions of the input vector. Then, LayerNorm regularization is carried out, and the features extracted by multiple heads are aggregated through FFN blocks to finally obtain the context feature *C* that represents the whole sequence.

The entire process can be summarized as follows: given a circRNA sequence with a batch size of m, the preliminary features $x^{\left(2\right)}$ and $x^{\left(3\right)}$ are extracted by CircRNA2Vec and EIIP descriptors, respectively. It is then encoded by an encoder (using a 1D convolutional neural network) as $z^{\left(2\right)}$and $z^{\left(3\right)}$, where the feature sequence length is n. Then, context variables $c^{\left(2\right)}$ and $c^{\left(3\right)}$ are extracted by improved transformer respectively, and cross-view mutual prediction is carried out. The loss functions are:

(5) $$L_{\mathrm{self}}=L_{s}^{23}+L_{s}^{32},$$

(6) $$L_{s}^{23}=-\frac{1}{n}\sum_{t=1}^{n}\mathrm{diag}\left(\text{log}\;\frac{\;\text{exp}\;\left({\left(W_{t}\left(c^{\left(2\right)}\right)\right)}^{T}z_{t}^{\left(3\right)}\right)}{\sum \;\text{exp}\;\left({\left(W_{t}\left(c^{\left(2\right)}\right)\right)}^{T}z_{t}^{\left(3\right)}\right)}\right),$$

(7) $$L_{s}^{32}=-\frac{1}{n}\sum_{t=1}^{n}\mathrm{diag}\left(\text{log}\;\frac{\;\text{exp}\;\left({\left(W_{t}\left(c^{\left(3\right)}\right)\right)}^{T}z_{t}^{\left(2\right)}\right)}{\sum \;\text{exp}\;\left({\left(W_{t}\left(c^{\left(3\right)}\right)\right)}^{T}z_{t}^{\left(2\right)}\right)}\right).$$

#### 2.3.2 Fine-tuning

After mutual prediction across the sequence of views, we get the trained RNA_Transformer. This allows him to learn the expression of the context of the overview from the sequence features. We then fine-tune the network for specific tasks to meet the needs of circRNA–protein binding site prediction. Specifically, we combine the features $z^{\left(\mathrm{all}\right)}$ encoded by the above three feature descriptors and input them into RNA_Transformer. Context information $c^{\left(\mathrm{all}\right)}$ of fusion features is extracted, processed by projection_head and normalized by softmax to obtain prediction label $\hat{y}$. Finally, using cross-entropy loss and training with only a very small number of real labels, excellent results can be obtained:

(8) $$\hat{\text{y}}=\frac{\;\text{exp}\;\left(\sigma \left(W_{p}\left(c^{\left(\mathrm{all}\right)}\right)+b_{p}\right)\right)}{\sum_{m}\;\text{exp}\;\left(\sigma \left(W_{p}\left(c^{\left(\mathrm{all}\right)}\right)+b_{p}\right)\right)},$$

(9) $$L_{f}=-\frac{1}{m}\sum_{i=1}^{m}\left[y_{i}\;\text{log}\;{\hat{\text{y}}}_{i}+(1-y_{i})\;\text{log}(1-{\hat{\text{y}}}_{i})\right].$$

As far as we know, this is the first time to apply the SSL algorithm to address the RNA–protein binding site prediction problem. Different from HCRNet and CircSSNN, the three feature descriptors we selected are relatively shallow algorithms and do not use DNABert’s large language model, which requires lower hardware resources and is easy to be widely used. Compared with previous supervised learning algorithms, it reduces the excessive dependence on actual labels. After representing sequences in agent task learning without using real tags, superior performance can be achieved with only a small number of tags depending on the final task.

## 3 Results and discussion

### 3.1 Experimental setup

In our experiment, the networks are trained by the Adam optimizer, where $\beta_{2}=0.99$, $\beta_{1}=0.9$, and weight_decay is set to 3e-4 and batchSize to 64. The optimizer’s learning rate is automatically controlled by the scheduling that comes with pytorch, where initial value is 3e-3. We employ a layer of RNA_Transformer and set dim to 400, heads to 8, and mlp_dim to 200.

### 3.2 Existing supervised algorithm performance

**Figure 3.**
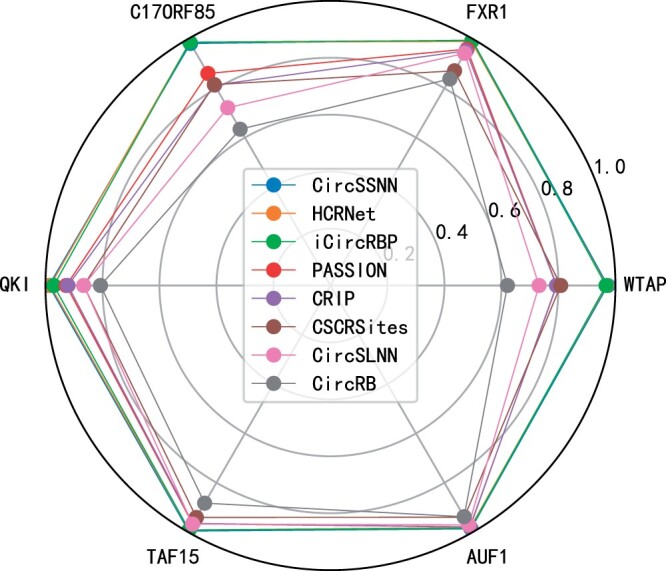
AUC discrimination performance obtained by eight existing supervised algorithms.

**Figure 4.**
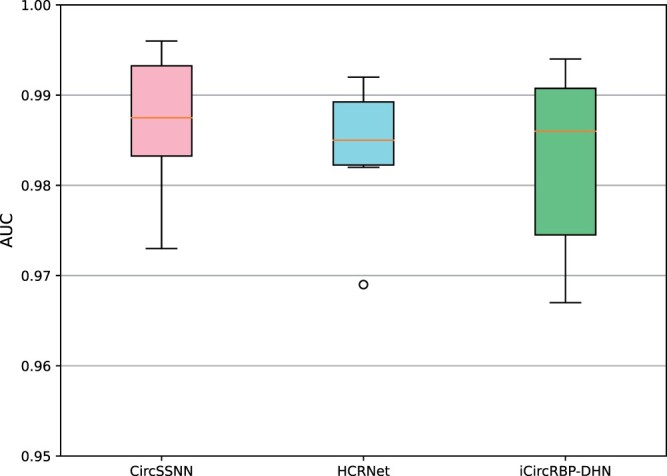
AUC performance obtained by the latest three supervised learning algorithms on six datasets.

We demonstrate the AUC performance achieved by eight existing supervised recognition algorithms on six circRNA–RBP datasets, as shown in Fig. 3. These include CircSSNN, HCRNet, iCircRBP-DHN, PASSION, CRIP, CircRB, CSCRSites, and CircSLNN. The dataset ratio is set to 8:2, based on the number of training and test samples as claimed in their respective papers. It can be seen from the picture that the latest algorithm CircSSNN has achieved nearly perfect performance, and HCRNet and iCircRBP-DHN are not much different from it. Since Fig. 3 cannot be well distinguished, we independently draw the results of these three algorithms on these datasets to draw box plots (Fig. 4). However, it should be noted that these algorithms require up to 80% of the training samples, i.e. 80% of the labeling labels obtained through biological experiments need to be invested in the algorithm for auxiliary learning, so as to guide the network to learn useful and easily distinguishable features. We know that the biological experiment analysis cost is high, the cycle is long, the efficiency is low, consumes a lot of human, material, and financial resources, which greatly limits the universality of the algorithm. Therefore, the algorithm’s dependence on labels should be reduced as much as possible to reduce the cost.

### 3.3 Our CircSI-SSL performance

**Figure 5.**
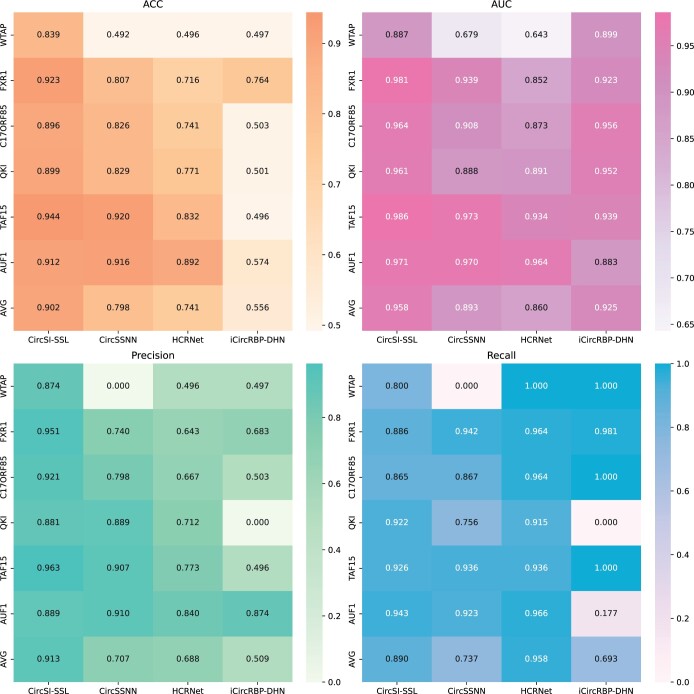
Performance comparison between CircSI-SSL and the latest three supervised algorithms in four indicators.

**Figure 6.**
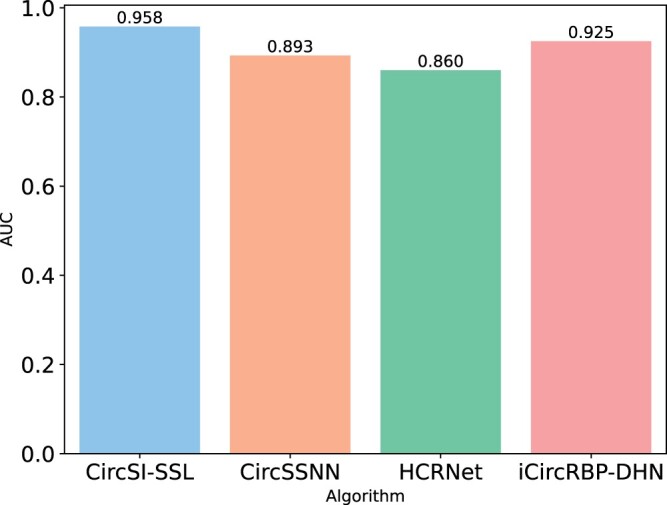
Average AUC performance comparison between CircSI-SSL and the latest three supervised algorithms on six datasets.

To validate the low dependency for labels and recognition effectiveness of our CircSI-SSL algorithm, we selected three algorithms with the best supervised performance, CircSSNN, HCRNet, and iCircRBP-DHN, and compared them with our algorithm under the premise of train:test = 1:9. The results are shown in Fig. 5. It can be seen that our algorithm has achieved remarkable performance on most datasets and indicators, but we also see that our algorithm is slightly lower than HCRNet in Recall index. The reason may be that when very little supervision information is involved in training, supervised algorithms tend to pay too much attention to individual indicators and failure to achieve overall performance. For example, HCRNet focuses on recall index, while ACC and Precision fail to achieve good results. In contrast, our CircSI-SSL achieves a balanced and excellent performance across all metrics. It can also be seen from the comprehensive index AUC that the algorithm in this article has the best comprehensive ability and has a wide application prospect. For the convenience of comparison, we visualized the average AUC of the algorithm on six datasets as Fig. 6. It can be intuitively seen that the algorithm in this article achieved the highest performance compared with other datasets, which was 3.3% higher on average and more than 5% higher on some datasets.

To further explore the relationship between the performance of the proposed algorithm and the amount of supervised information introduced, it is proved that the proposed algorithm can achieve stable performance under the condition of very few training samples. We conducted a step test according to the training samples from 1 to 9. Ratio was used to represent the ratio between the training set and the test set. The AUC performance obtained was shown in Table 1. We can see that in general, the algorithm has learned easily distinguishable features under the sample ratio of 1:9, and achieved excellent classification performance. With the continuous increase of training samples, the performance of the algorithm can maintain a certain increase, but the difference is not much compared with the initial. This fully indicates that the cross-view prediction task based on SSL has trained the RNA_Transformer feature extractor and learned enough contextual features to represent the entire sequence. Only a very small number of samples are required to fine-tune for subsequent recognition tasks.

**Table 1.** AUC performance of CircSI-SSL algorithm under different training sample proportions.

Ratio	WTAP	FXR1	C17ORF85	QKI	TAF15	AUF1	AVG
1:9	0.887	0.981	0.964	0.961	0.986	0.971	0.958
2:8	0.914	0.980	0.962	0.964	0.986	0.970	0.963
3:7	0.913	0.984	0.962	0.967	0.987	0.973	0.964
4:6	0.926	0.987	0.963	0.964	0.989	0.972	0.967
5:5	0.938	0.983	0.961	0.968	0.988	0.973	0.968
6:4	0.943	0.985	0.960	0.969	0.991	0.977	0.971
7:3	0.938	0.983	0.959	0.971	0.990	0.975	0.969
8:2	0.978	0.989	0.956	0.970	0.989	0.979	0.977

### 3.4 Ablation analysis

**Figure 7.**
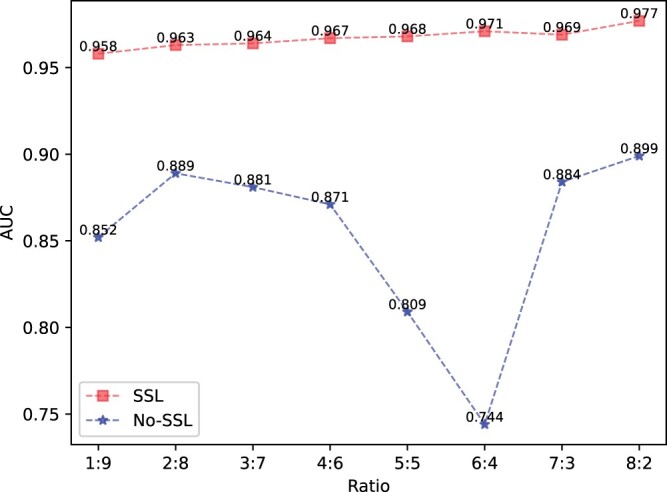
Average AUC performance with and without SSL across six datasets.

In this section, we conduct an ablation analysis to demonstrate that the improved performance of our algorithm is a direct result of the SSL task we designed. The AUC performance obtained by the CircSI-SSL algorithm on these datasets is presented in Table 2, where fine-tuning based on real labels is performed directly without cross-view sequence prediction task. It is evident that when no proxy task is performed, the algorithm performance drops off a cliff, with an average decline of about 10%, as shown in Fig. 7. In particular, there is also an extreme AUC performance of 0.5. This is sufficient to show that it is necessary to conduct self-supervised tasks, to learn the overall expression of the sequence from the data (without labels), and thus to significantly improve subsequent classification tasks with only a few labels.

**Table 2.** AUC performance of CircSI-SSL algorithm (without self-supervision task) under different training sample proportions.

Ratio	WTAP	FXR1	C17ORF85	QKI	TAF15	AUF1	AVG
1:9	0.881	0.883	0.941	0.866	0.888	0.653	0.852
2:8	0.876	0.895	0.944	0.838	0.931	0.848	0.889
3:7	0.878	0.923	0.941	0.923	0.913	0.711	0.881
4:6	0.885	0.904	0.936	0.926	0.831	0.744	0.871
5:5	0.918	0.938	0.733	0.673	0.915	0.673	0.809
6:4	0.907	0.500	0.945	0.703	0.909	0.500	0.744
7:3	0.902	0.898	0.940	0.934	0.853	0.775	0.884
8:2	0.930	0.886	0.946	0.873	0.836	0.922	0.899

### 3.5 Transplant analysis

**Figure 8.**
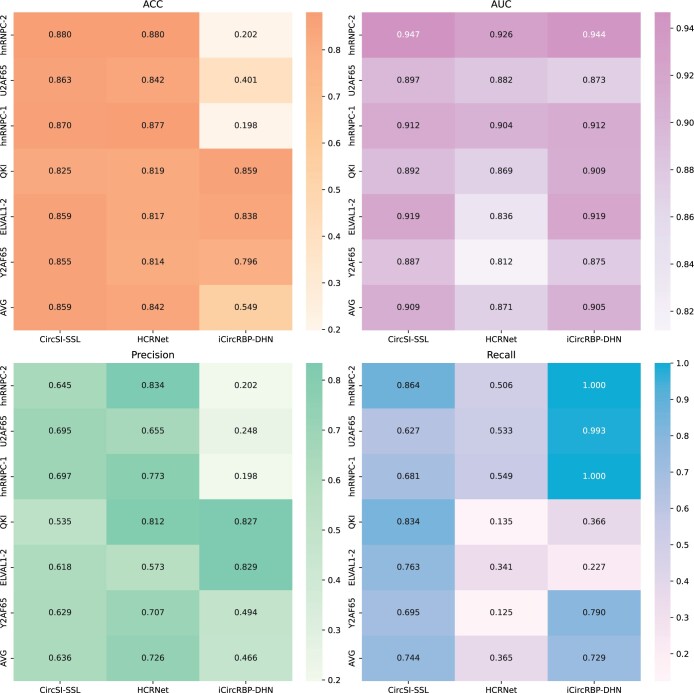
Comparison of transplant performance on linRNA datasets.

To further demonstrate the advantages of the proposed algorithm in more aspects, we transplanted the circSI-SSL algorithm originally designed for circRNA into the binding protein prediction task of linRNA without any network modification and with consistent hyperparameters. In the performance comparison between the six widely used linRNAs and several supervised algorithms as shown in Fig. 8 below, the ratio of training set to test set is still 1:9. Remarkably, the proposed algorithm achieves the best overall performance without any task-oriented tuning. In Fig. 8, we can see that although iCircRBP-DHN also obtained a good average AUC value, it can also clearly see huge fluctuations in ACC, Precison, and Recall, which are separate indicators. HCRNet algorithm is relatively stable, but its performance on Recall index is poor. In the case of a very small number of training datasets put into training, the performance of the above two in each indicator is not balanced, and the overall good performance is not achieved. Therefore, supervised learning algorithm is not a good choice when there are only a few labeled samples. In contrast, the algorithm in this article achieves the overall optimal performance, even in such a harsh environment.

## 4 Conclusion

In this article, we propose the novel CircSI-SSL framework for circRNA–RBP site recognition tasks based on SSL. By designing a cross-view sequence prediction task, the algorithm can learn the overall representation of the sequence in an unsupervised manner, and significantly enhance subsequent RBP identification performance with only a small amount of supervised information. Based on the improved Transformer network RNA_Transformer in this article, the framework extracts sequence context features from multiple views to characterize the sequence. By designing reasonable and effective proxy tasks, along with a stable and efficient network architecture, significant improvements were achieved with only a small amount of supervised information on the widely used six circRNA datasets and six linRNA datasets compared to supervised learning algorithms.

In short, the CircSI-SSL algorithm based on SSL has good identification performance, expansion performance, and wide application range, only a small amount of label information can significantly improve the recognition performance. It is a very competitive tool for circRNA–RBP binding site identification.
